# The Evolution of Two-Component Systems in Bacteria Reveals Different Strategies for Niche Adaptation

**DOI:** 10.1371/journal.pcbi.0020143

**Published:** 2006-11-03

**Authors:** Eric Alm, Katherine Huang, Adam Arkin

**Affiliations:** 1 The Virtual Institute for Microbial Stress and Survival, Berkeley, California, United States of America; 2 Department of Civil and Environmental Engineering, Massachusetts Institute of Technology, Cambridge, Massachusetts, United States of America; 3 Department of Biological Engineering, Massachusetts Institute of Technology, Cambridge, Massachusetts, United States of America; 4 Physical Biosciences Division, Lawrence Berkeley National Laboratory, Berkeley, California, United States of America; 5 Department of Bioengineering, University of California Berkeley, Berkeley, California United States of America; University of California San Diego, United States of America

## Abstract

Two-component systems including histidine protein kinases represent the primary signal transduction paradigm in prokaryotic organisms. To understand how these systems adapt to allow organisms to detect niche-specific signals, we analyzed the phylogenetic distribution of nearly 5,000 histidine protein kinases from 207 sequenced prokaryotic genomes. We found that many genomes carry a large repertoire of recently evolved signaling genes, which may reflect selective pressure to adapt to new environmental conditions. Both lineage-specific gene family expansion and horizontal gene transfer play major roles in the introduction of new histidine kinases into genomes; however, there are differences in how these two evolutionary forces act. Genes imported via horizontal transfer are more likely to retain their original functionality as inferred from a similar complement of signaling domains, while gene family expansion accompanied by domain shuffling appears to be a major source of novel genetic diversity. Family expansion is the dominant source of new histidine kinase genes in the genomes most enriched in signaling proteins, and detailed analysis reveals that divergence in domain structure and changes in expression patterns are hallmarks of recent expansions. Finally, while these two modes of gene acquisition are widespread across bacterial taxa, there are clear species-specific preferences for which mode is used.

## Synopsis

Pathways containing histidine protein kinases (HPKs) represent a key mechanism for signal transduction, especially in bacteria. These systems help cells to sense and respond to their environment by detecting external cues and effecting internal responses such as changes in gene expression. As such, they are believed to play a key role in niche adaptation, yet their evolution is difficult to study due to the large number of paralogous subfamilies. This work extends previous large-scale gene evolution studies by considering complex paralogy relationships, and uncovers an abundance of horizontal transfers, gene duplications, and domain shuffling that have marked the evolutionary history of HPKs. An important finding of this study is qualitative differences between the main strategies for acquiring new HPKs (horizontal gene transfer and gene duplication). Hallmarks of the latter process include domain shuffling and the generation of “orphan” HPKs not co-transcribed with a cognate response regulator.

## Introduction

Bacteria change their physiological behavior according to signals detected in their environment. Typically, these changes are reflected in the alteration of gene expression patterns. These changes can be the result of action by a number of different types of signaling proteins, including histidine protein kinases (HPKs) and their cognate response regulators (RRs), methyl-accepting chemotaxis proteins, di-guanylate and adenylate cyclases, and serine/threonine/tyrosine protein kinases, as well as individual transcription factors or “one-component” signal transduction proteins [1,2]. Of these various protein families, HPKs are among the most abundant, and historically have been regarded as the primary mechanism for signal transduction in bacteria [3].

HPKs, and more generally signal transduction proteins, are thought to play a major role in the adaptation of bacteria to new or changing environments. Consistent with this hypothesis, those bacteria that have the largest complements of signaling proteins generally tend to be bacteria with complex lifestyles such as *Myxococcus xanthus,* those that are found ubiquitously in varied environments such as *Pseudomonas,* or bacteria with numerous alternative metabolic strategies such as various δ- and ɛ-proteobacteria [2,4]. By contrast, few HPKs have been identified in the reduced genomes of parasitic bacteria, which likely have a relatively constant external environment.

While these signal transduction systems are thought to be a key part of the adaptive evolution of bacteria, few details are known about this process. In this study, we investigated the distribution of HPKs in sequenced bacterial genomes to address some fundamental questions: (1) What fraction of HPKs in a given genome represents newly acquired/ancient genes? (2) What are the evolutionary processes that give rise to new HPKs? (3) Do newly acquired HPKs sense similar signals or do they evolve new functionality?

We looked specifically at genes that entered into each lineage recently, making the logical assumption that recent additions are more likely to provide insight into the evolutionary basis of niche adaptation. Identifying recent acquisitions in a background of multiple paralogs is a difficult task. We describe a BLAST-based procedure for classifying and establishing the age of HPK domains. This procedure is derived from previous gene presence/absence studies by our group and others [5–7]. We based our phylogenetic analysis on the histidine kinase domain of each HPK only, allowing us to follow changes in the structure of the signaling domains (domain shuffling) that generally lie upstream (N-terminal) from the kinase domain. The phylogenetic inference procedure described requires an accurate species phylogeny, which we inferred from a concatenated gene profile including 15 ubiquitous bacterial genes without obvious paralogs (Table S1).

We used the gene histories inferred by this procedure to estimate the relative contribution of horizontal gene transfer (HGT) and gene duplication events to the evolution of new HPKs in each genome. We observed that some genomes acquired new HPKs primarily via HGT, while others relied mainly on lineage-specific expansion (LSE) of existing gene families. A closer look at genes acquired via these two mechanisms revealed differences in the extent to which upstream signaling domains and cognate RRs were conserved as a result of each process, with HGT being more likely to preserve pre-existing relationships than gene duplication. We investigated one such HPK expansion in Desulfovibrio vulgaris in greater detail, and describe specific examples of LSE-associated domain shuffling. We further looked to functional genomic data and confirmed that these new HPKs have distinct gene expression profiles, suggesting novel functional roles.

### Inferring Gene Histories

In this study, we identified nearly 5,000 HPKs from 207 genomes. We parsed each of these HPKs into domains (signaling domains of various types, HPK domains, and RR domains in some cases), and analyzed the evolution of the HPK domain from each gene using an approach based on pairwise BLASTp scores. In this way, we identified domains that were more similar to genes in the same genome than to genes in other genomes as the likely result of LSE. For domains that were more closely related to genes in distant genomes than those in more closely related genomes, we inferred HGT as a likely explanation. Finally, we identified a small number of subfamily “Birth” events when a particular group of genes was found in a narrow range of species. These genes are sometimes referred to as “ORFans” [6].

**Figure 1:**
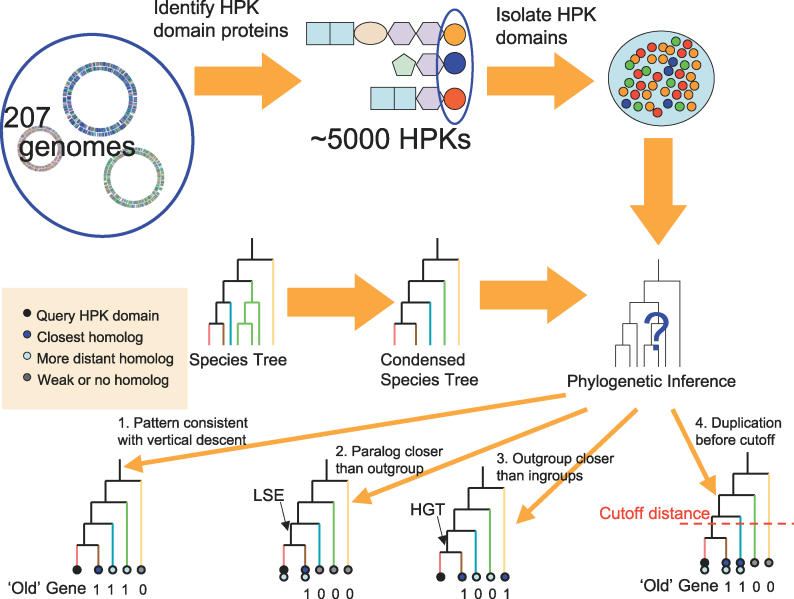
Overview of Approach An overview of our phylogenetic inference procedure is given. We look only at histidine kinase domains from HPKs, and compare the distribution of these to the species tree. When homologs in distant outgroups are more distantly related, we infer simple vertical descent. Paralogs and distances that contradict species phylogeny result in our inference of gene duplication or horizontal transfer. Only events (such as duplication or transfer) that occurred more recently than the cutoff as described in the Methods are considered. Four hypothetical cases are shown, and each is labeled as present/absent (“1” or “0”) from each outgroup according to the procedure described in the Methods.

An overview of our approach is given in Figure 1. A complete outline of our algorithm for inferring domain histories is given in the Methods section, but a few key details are worth noting here. First, our approach relies on a species tree, which we constructed using a set of ubiquitous single-copy genes. This species tree is then condensed into a set of “outgroups” at increasing evolutionary distances from the species of interest [5,6,8]. If the best hit of a HPK domain to a distant outgroup is closer than its best hit to a more closely related outgroup, then it is considered to be “absent” from the closer outgroup. When a gene is absent from two or more consecutive outgroups, implying multiple deletion events that could be alternatively explained by a single HGT event, the HGT event is inferred as more parsimonious, but *only if* the oldest outgroup branching after the presumed HGT diverged more recently than the phylogenetic cutoff distance for “recent” events (see Methods). As noted later in the text, we reproduced the main findings described in the Results with a more conservative definition of HGT (absence from three consecutive outgroups) with nearly identical results. When the distance to a HPK domain within the same genome was closer than to the best hit from an outgroup, then a duplication (LSE) event was inferred, but *only if* the outgroup diverged more recently than the phylogenetic cutoff.

Further information can be inferred about the time of each HGT or LSE event based on the set of genomes containing the HPK domain (e.g., was the HPK domain duplicated before or after the divergence of its host species with some other species?). We have been careful throughout this discussion to refer to the HPK domain rather than to the HPK itself—this is to emphasize the fact that we are inferring histories for only the HPK domain of signaling proteins, which (as shown in Results) can be very different from the evolutionary histories of associated signaling domains that are usually found within the same gene.

### Key Assumptions

Our approach makes several key assumptions. First, we rely on an accurate species tree. Our species tree compares quite favorably with other published phylogenies [9,10], and is available as part of Dataset S3. Further, we restrict our analysis to nodes with high bootstrap support. In addition, there is no real consensus among researchers as to the topology of the deepest branches, so even well-supported branching patterns according to concatenated gene trees may disagree with trees produced using other methods. We restrict our analysis to relatively recent evolutionary events, in part to avoid complications that result from uncertainty in these deepest branches. We also make the key assumption that under a model of vertical inheritance, the best BLASTp hit of each domain should be to its most closely related ortholog. This plausible assumption can be violated for a number of reasons, including unequal evolutionary rates among different lineages. To minimize problems of this type, we impose a stronger cutoff to conclude “absence” of a gene from an outgroup based on a more similar homolog in a distant outgroup: the BLASTp score to the more distant outgroup must be 20 greater (in raw bit score) than the score to the closer outgroup. All of the raw scores used in this analysis are available for browsing online at http://microbesonline.org/hpk. Because we require domains to be “absent” from two or three consecutive well-supported outgroups, and focus only on recent evolutionary events, we feel that it is very unlikely that this assumption is violated in a significant enough number of cases to affect clear trends reported in this study.

## Results

### An Overview of HPKs across Bacteria

**Figure 2:**
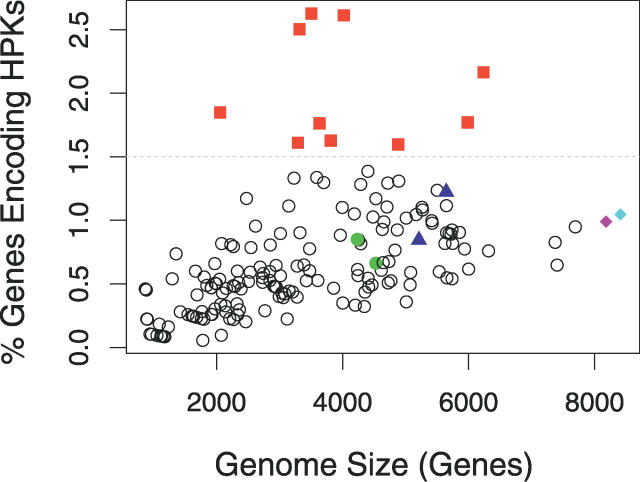
HPK Content versus Genome Size The percent of each genome is plotted as a function of genome size. As reported in previous studies, there is a roughly linear correlation. Highlighted in colored symbols are several groups of genomes described in the text: genomes coding a high (≥1.5%) fraction of HPKs—red squares; the model organisms, E. coli and *B. subtilis—*green circles; genomes with a high number of HGT events*—*blue triangles; Bradyrhizobium japonicum (high number of both HGT and LSE genes)*—*pink diamond; and Streptomyces coelicolor (high percentage of LSE genes)*—* turquoise diamond.

The fraction of HPKs coded by a given genome is known to scale roughly with the size of the genome, as shown in Figure 2 (an even better correlation is seen if all signaling proteins in a genome are considered [2]). We wanted to identify and investigate genomes that had particularly high numbers of HPKs to see if we could identify their origin. Did these genomes duplicate existing HPKs, acquire large amounts through HGT, or did they simply lose fewer “old” HPKs than other genomes? Several different types of genomes were chosen as examples for more in-depth study throughout this manuscript (and details for all are available in Dataset S1). First, we chose organisms in which more than 1.5% of the genome codes for HPKs (red squares in Figure 2). We also targeted two genomes that had the largest numbers of genes acquired by HGT, Ralstonia solanacearum and Pseudomonas syringae (blue triangles in Figure 2), and one genome in which nearly every new HPK gene was acquired through LSE, Streptomyces coelicolor (pink diamond in Figure 2). We chose Bradyrhizobium japonicum (turquoise diamond in Figure 2) because it includes large numbers of new HPKs acquired through both HGT and LSE, so we could compare these two processes in a single genome. Finally, we included the model organisms Bacillus subtilis and *Escherichia coli,* in which HPKs are the most well-studied experimentally (green circles in Figure 2).

### Different Species Rely on Different Mechanisms for Acquiring New HPKs

**Figure 3:**
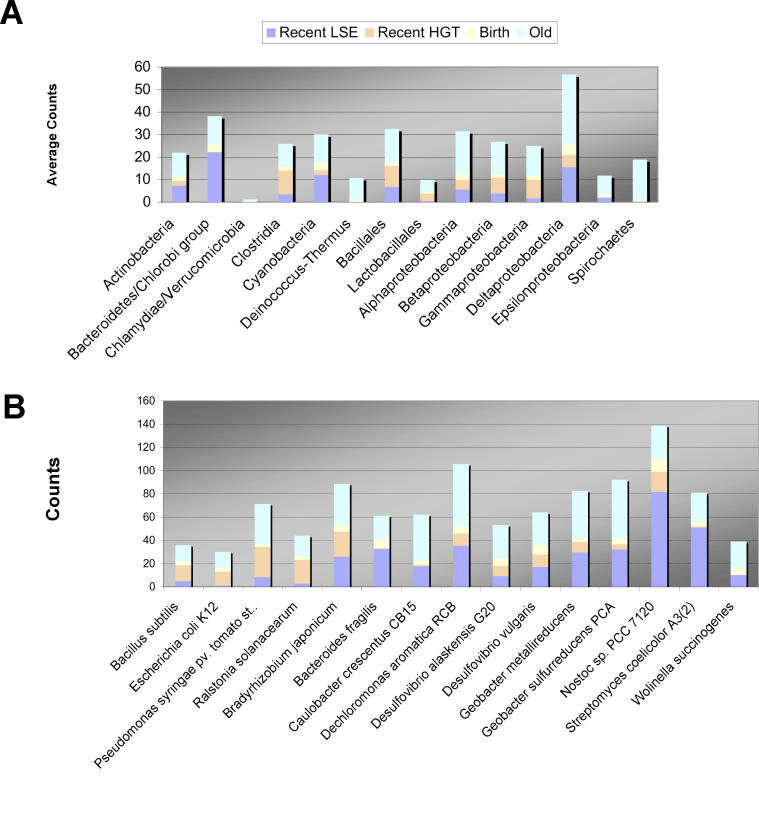
Summary of Evolutionary Events The number of events inferred for different bacteria is summarized in this figure. (A) Average numbers for the major taxonomic groups used in this study. (B) Specific numbers for targeted genomes (those with colored symbols in Figure 1).

Figure 3 summarizes the quantitative results of our phylogenetic analysis across all bacteria (Figure 3A) and individually for each of our targeted genomes (Figure 3B). New HPKs are common across the bacteria; however, different genomes encode different numbers of new genes. Bacteria in the δ- and ɛ-groups of the proteobacteria contain particularly high numbers of recently acquired HPKs.

**Figure 4:**
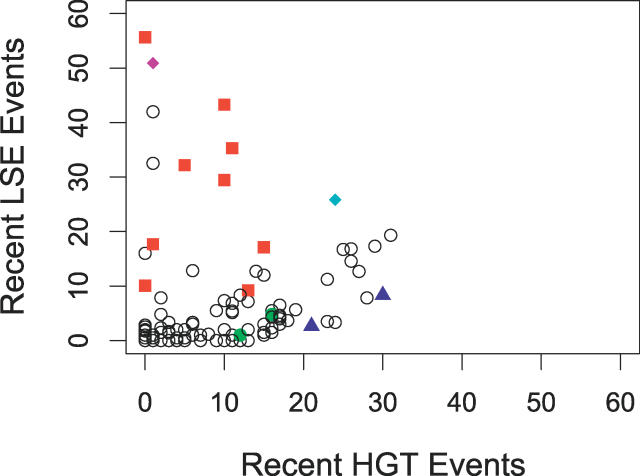
LSE Events versus HGT Events The number of LSE and HGT events for each genome are shown. Colored symbols correspond to the genomes identified in Figure 1. Note the position of the red squares well above the *x*-axis.

The number of new HPKs arising through HGT or LSE is quite variable across different phylogenetic groups, as shown in Figure 4. In some genomes, such as E. coli and *R. solanacearum,* recent gene duplications are rare. HGT, on the other hand, accounts for nearly all of the recently acquired HPKs in these genomes. For others, such as D. vulgaris and Geobacter sulfurreducens, LSE accounts for the majority of recently acquired HPKs. Streptomyces spp. are known for their propensity for gene duplication [11], and their new HPKs result almost exclusively from LSE. The mechanism of gene duplication in S. coelicolor is qualitatively unlike that of other genomes in this study; this point is discussed in greater detail in following sections.

The question of why different genomes have different preferences for HGT or LSE as a means of acquiring new signaling proteins is not obvious, but we did find that genomes with unusually large numbers of HPKs relative to their genome size tend to have accumulated those HPKs via LSE. The fraction of HPKs in a genome involved in recent LSE correlates strongly with the total number of HPKs in that genome (ordinary least squares linear regression: r = 0.74, *p* < 10^−15^), while the fraction involved in a recent HGT event does not (r < 0.1, *p* = 0.93). In fact, all of the genomes that devote at least 1.5% of their genes to encoding HPKs (Nostoc sp. PCC 7120, Geobacter spp., Desulfovibrio spp.*, Wolinella,* and *Dechloromonas*)*,* which are highlighted in red in Figure 2, have major LSEs.

In addition, while all of these genomes (excluding *Nostoc*) are dissimilatory sulfur- or sulfate-reducing bacteria, and many are more closely related δ-proteobacteria, they do not necessarily contain the same expansions. For example, the *Geobacter* lineage contains a large expansion in “type 3” HPKs (using the standard nomenclature defined in [12]), while the *Desulfovibrio* lineage contains an expansion in type 4 HPKs. A further expansion of the “hybrid” type 1b family (including both histidine kinase and RR domains in the same protein) is seen only in *D. vulgaris,* and not in its close relative D. alaskensis G20 (also known as D. desulfuricans G20). Thus, while the propensity for gene duplication may be an inherited trait among these broadly related δ-proteobacteria, the major expansions in each organism are not necessarily shared.

### LSE Disrupts HPK–RR Operon Structure

**Figure 5:**
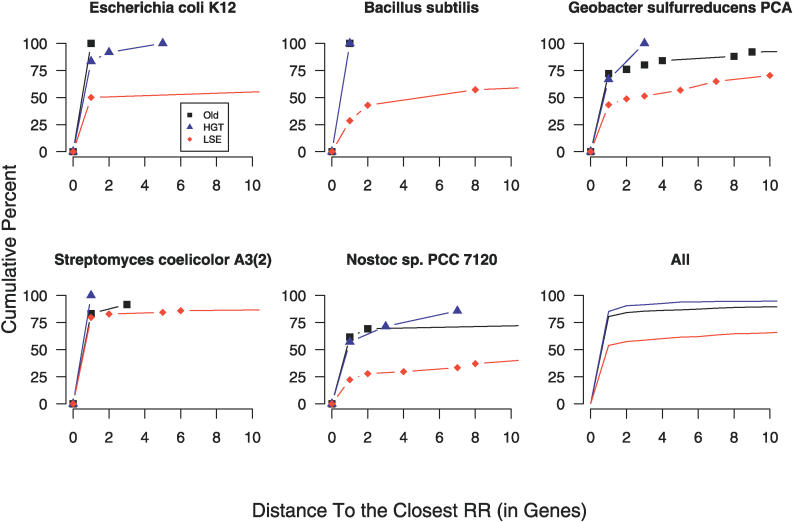
Proximity of Different Classes of HPKs to RRs Shown is the cumulative percentage of HPKs of each type that have RRs within the distance on the chromosome specified by the *x*-axis. The different gene types shown are: old HPKs*—*black squares (Old); HGT genes without recent paralogs*—*blue triangles (HGT); and HPKs with recent paralogs*—*red diamonds (LSE). In the bottom right panel, an average over all genomes is shown. In general, and for most specific cases (excepting *Streptomyces*), horizontally transferred genes are observed to have a much higher fraction of RRs in close genomic proximity. “Hybrid” HPKs, which have RRs in the same ORF as the HPK, were excluded from this analysis. Only genes that are not believed to have undergone duplication within a lineage are used in the HGT group. Lines stop when cumulative percentage equals 100%.

Compared to new HPKs acquired through HGT, HPKs resulting from LSE are less likely to have coevolved with their cognate RRs in a single duplication event. Figure 5 shows the distance of new HPKs to response regulators. The data shown do not include “hybrid” HPKs (HPK and RR domains in the same polypeptide chain), which can bias analysis due to their apparent propensity for LSE, and since there is already a RR in the same gene by definition. Averaged over all genomes or taken individually for particular genomes, the trend is clear—LSE genes are much more likely to be present as “orphans,” separated from their cognate RRs in the genome. *S. coelicolor* is an unusual exception to this trend, as it has high numbers of RRs in the immediate proximity of duplicated HPKs. To confirm that operons were the most likely explanation for this genomic proximity between HPKs and RRs, we also compared these different classes of HPKs to operon predictions that have been validated across a wide range of species [13,14], and observed the same trend: 77% of HGT HPKs had a co-operonic RR, compared to 69% for “old” HPKs, and only 42% for LSEs.

**Figure 6:**
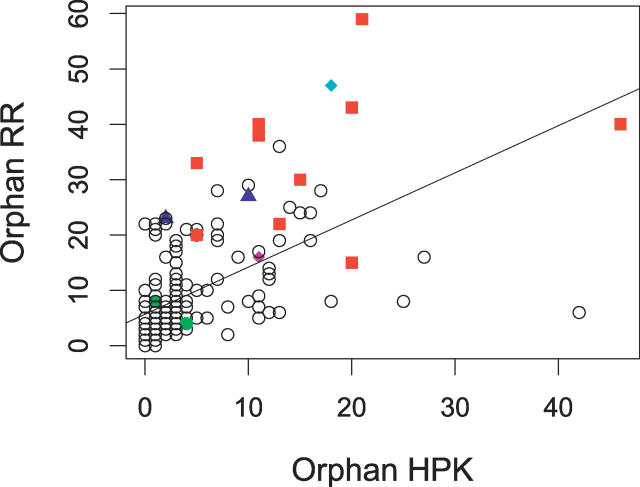
Coevolution of Orphan HPKs and RRs The number of “orphan” HPKs is plotted versus the number of “orphan” RRs. A moderate but highly significant linear correlation is observed (ordinary least squares linear regression: r = 0.57, *p* < 10^−15^).

This separation between HPK and RR evolutionary events suggests that these novel LSEs may be more likely to engage in crosstalk. This is certainly the case for the sole LSE in *B. subtilis,* which is made up of the *kin* regulators of sporulation. *KinA–E* are thought to integrate signals into a common downstream target based on their approximately equal affinity for the regulator *Spo0F* [15]. By contrast, the sole recent duplication in *E. coli,* resulting in the *NarQ/NarX* genes, avoids crosstalk as each HPK ties into a distinct regulator (*NarP* and *NarL,* respectively) [16,17]. A recent study by Laub and coworkers in Caulobacter crecentus also found little evidence for physiologically relevant crosstalk among HPKs [18]. If crosstalk does not play a large role in general, we would expect to see that the number of “orphan” RRs (not in proximity to a HPK) would generally correlate with the number of “orphan” HPKs (not in proximity to a RR). Figure 6 shows that this trend largely holds across the species examined, though many species show large deviations. We suspect that while some crosstalk may indeed occur, the results from Laub and coworkers are likely to apply to some extent even across species with large numbers of duplications. Experimental work in these species will be necessary to answer this important question.

In some cases, we observed that one or a small number of HPKs in an expansion are positioned in operons with RRs. Although beyond the scope of this study, an interesting hypothesis is that these HPKs may be the progenitors of the expansions. For example, *NarQ* is co-operonic with *NarP,* while its duplicate *NarX* is transcribed separately from its cognate regulator, *NarL.* We also observed that the small numbers of HGT genes in genomes with large LSEs are likely to have cognate RRs nearby. This may not only reflect the fact that HPKs are likely to transfer into a genome with their cognate regulators, but also that those HPKs near their cognate regulators make better candidates for transfer out of a genome than their paralogous copies. Indeed, we recently reported a relationship between operons and HGT [5]. We found that nearly 50% of new HGT genes in E. coli were acquired with another gene as part of a horizontally transferred operon.

### Domain Shuffling Often Accompanies LSE

**Figure 7:**
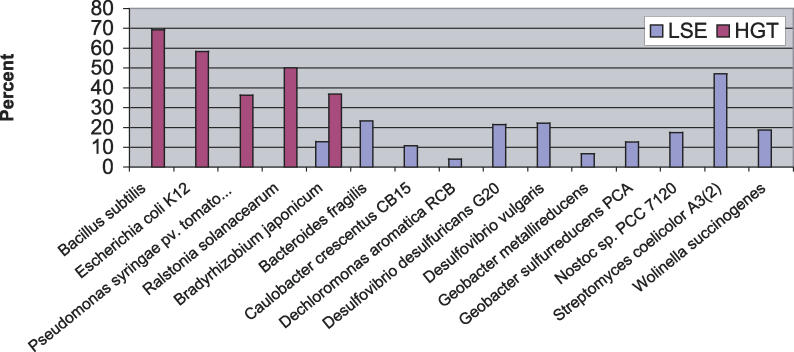
Extent of Domain Shuffling in Different Classes of HPKs The fraction of HPKs with identical upstream domains to either their inferred HGT partners (red bars), or to their closest paralog (blue bars) in the case of LSE. Only genes that are not believed to have undergone duplication within a lineage are used in the HGT group. B. japonicum, which has a significant number of genes classified as HGT and LSE, is shown twice.

HPKs generated by LSE also display more novel variation in their (usually N-terminal) sensory domains than those acquired horizontally. Across all genomes, 47.4% of horizontally transferred HPKs retain a set of upstream signaling domains identical (in both domain type and linear order) to their inferred HGT partner, whereas only 29.1% of recent duplications retain the same domain structure as their closest paralog. In fact, for expansions that include five or more proteins, only 19.9% of closest paralogs had an identical set of upstream domains. Figure 7 shows results for individual genomes. The fraction of HGT genes with conserved upstream domains are shown for those genomes rich in HGT events, and the fraction of LSE genes with conserved upstream domains are shown for those genomes rich in LSE. For *B. japonicum,* which contains a mixture of both types of new genes, both numbers are shown. As a control, we also considered a more stringent definition of HGT requiring genes to be absent from three consecutive outgroups. Using this more stringent definition, 47.3% of horizontally transferred HPKs were found to retain an identical series of upstream signaling domains, which is nearly identical to the 47.4% obtained from the less-stringent definition. In addition, we considered the possibility that horizontally transferred HPKs might have a tendency not to include any additional signaling domains, and therefore may be identical trivially. We found that only ten of our 420 HGT genes lacked any signaling domains, supporting our original conclusions.

These results are particularly striking since the horizontally transferred HPK domains are on average less similar (lower BLASTp sequence identity) than paralogous domains. These results are also encouraging because our evolutionary inference methods are based only on the similarity of the histidine kinase domain of each HPK, and the high rate of similarity of these upstream signaling domains between putative HGT partners supports the accuracy of our approach. In these results, we considered genes derived from an HGT event followed by a duplication event in the totals for duplicates, but not when computing the totals for HGT, as it is not possible using our method to determine which of the resulting paralogs is more likely to have retained the ancestral state of signaling domains.

A notable outlier in Figure 7 is worth mentioning: S. coelicolor contains the largest fraction of new genes acquired by LSE of all the genomes we studied, yet a large fraction of these genes contain an identical set of upstream signaling domains. In addition, as reported in a previous section, LSEs in this species tend to involve duplications that preserve HPK–RR pairings. These qualitative differences may reflect an enhanced capability of this genome to duplicate regions of its linear chromosome, a process that has been proposed previously based on genome sequence analysis [11].

Taken together, the results presented in this section suggest different roles for HGT and LSE in HPK evolution. While both mechanisms contribute to the diversity of signaling systems, LSE is accompanied by rearrangements in domain structure as well as by independent evolution of HPK and RR genes. By contrast, organisms such as *B. subtilis, E. coli,* and P. syringae appear to acquire new HPKs via horizontal transfer of intact two-component systems. These consumers of preexisting genetic diversity are less likely to contain completely novel domain structure, and are more likely to include HPKs in proximity to their cognate RR. Individual genomes appear to have very different preferences for HGT or LSE. LSE is the dominant force in species that are the most highly regulated (those with the highest proportion of genes coding for HPKs), whereas HGT appears to be dominant, for example, in the well-studied model systems E. coli and *B. subtilis.*

### Anatomy of an LSE

**Figure 8:**
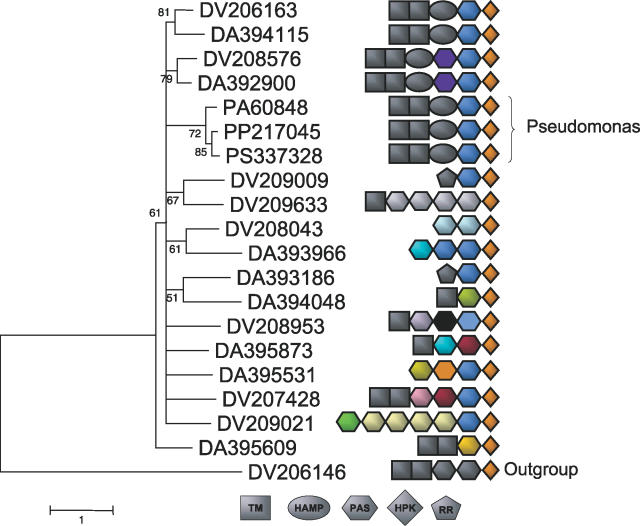
Domain Shuffling in a Desulfovibrio spp. Expansion The domain structure of genes in a large LSE in the *Desulfovibrio* genus is shown. In addition, three similar proteins identified in *Pseudomonas* species are shown, which are likely the result of HGT. Genes are identified by their species name and their accession number in the MicrobesOnline database (http://microbesonline.org) for easy reference (DV refers to genes present in D. vulgaris and DA refers to genes present in D. alaskensis G20). Each domain corresponds to a branch of the TREE-PUZZLE phylogenetic tree (of only the HPK domains) shown at left. Each PAS domain is colored according to sequence homology (as inferred by BLASTp), and domains with the same color comprise subfamilies of closely related domains. While upstream domains are generally shuffled, each gene shown contains a PAS domain immediately preceding the conserved HPK domain. Moreover, this PAS domain is largely conserved among paralogs at the sequence level, while more N-terminal domains are not. Interestingly, the *Pseudomonas* gene, which we infer to be involved in a horizontal transfer event, has a set of signaling domains identical to one of the *Desulfovibrio* copies, suggesting a likely donor–acceptor pair, and highlighting the qualitative difference in genes acquired by HGT and LSE.

To better understand the structure of an LSE, we investigated a single expansion in the two sequenced *Desulfovibrio* species. Several striking features are present in the expansion depicted in Figure 8. First, the diversity in the upstream signaling domains is obvious in the nonorthologous pairs of HPKs (some likely orthologs between D. vulgaris and *D. alaskensis* are shown, and have a similar set of upstream domains). Second, there was likely a HGT event between *Desulfovibrio* and *Pseudomonas* (probable orthologs from three *Pseudomonas* species are shown in the tree), which conserved an upstream domain structure (TM-TM-HAMP-PAS-HPK). This domain structure is identical between the *Pseudomonas* species and one of the members of the *Desulfovibrio* expansion, which we postulate served as the donor or acceptor. Third, many of the upstream signaling regions contain repeated domains, but only some of these are noticeably more similar in sequence than other pairs. Thus, rearrangements involve domains that are acquired from distant sources or domains that have been subject to more rapid evolution than HPK domains. Finally, there appears to be a mixture of proteins with and without predicted transmembrane domains, implying that the same basic architecture can support both kinds of signaling mechanisms. Further domain shuffling may also be happening at the level of the extracellular sensory regions not detected by our sequence profiles.

A close inspection of Figure 8 reveals a pattern in the signaling domain architecture of this expansion: every gene observed has a PAS domain immediately upstream of the HPK domain. Upon closer inspection, we found that this domain is not only conserved in its placement relative to the HPK domain, but is also highly conserved at the sequence level in most of the genes in this family, with clear sequence homology detectable even in the *Pseudomonas* species. This implies that domain architecture is not completely plastic. Instead, there appear to be “rules” for constructing new functional paralogs, and certain domains may be necessary to preserve optimal activity. Similarly, several expansions in *Nostoc* consist of a conserved set of core domains preceded by a variable upstream (N-terminal) region. Some other expansions we studied did not display such obvious patterns of domain architecture. The role of these conserved and nonconserved domains and their mechanism of interaction remains a key open question.

### New Functional Roles for Recently Duplicated Paralogs

**Figure 9:**
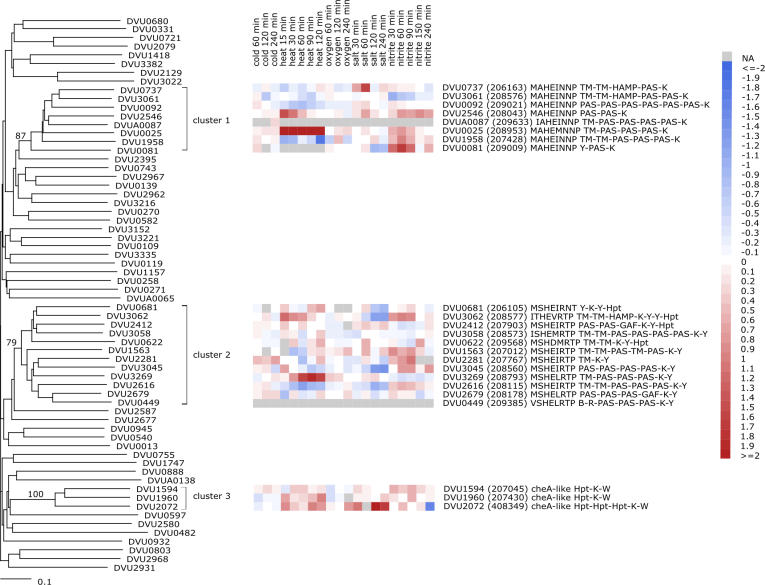
Gene Expression of D. vulgaris Expansions Gene expression profiles across a compendium of experimental stress response conditions (NaCl, heat shock, cold shock, nitrite, and oxygen) were monitored using DNA microarrays, and shown next to a phylogenetic NJ tree (with 1,000 bootstraps, generated using the MEGA3 software package [28]) of all HPK domains in *D. vulgaris.* Blue colors indicate down-regulated genes (relative to unperturbed cells), and red colors indicate up-regulated genes. No significant excess correlation in gene expression was observed for genes within each cluster (compared with randomly chosen pairs of genes) using a Student's t-test to compare mean correlations or the Kolmogorov-Smirnov test to compare distributions. The domain structure of each gene in the three LSEs is shown to the right. Gene names are provided for all genes, and MicrobesOnline accession numbers are provided in parentheses for genes in each of the major clusters for comparison with Figure 8. Bootstrap values are provided for each of the major clusters, and the amino acid sequence of the “H-Box” motif for genes in each cluster is shown. A more detailed description of the experiments performed is given in Methods.

LSEs contain a diversity of upstream signaling domains, suggesting that they might respond to different environmental signals. To test this hypothesis, we analyzed microarray data collected for D. vulgaris under a variety of stress-response conditions to determine whether paralogs had similar expression patterns. Surprisingly, Figure 9 reveals no detectable similarity in gene expression patterns among close paralogs, nor overall similarity within the two *Desulfovibrio*-specific clusters of HPKs. The correlations of gene expression profiles for closest paralogs is not significantly different from those observed between random pairs of genes as measured by the Student's *t* or Kolmogorov-Smirnov tests (as implemented in the R statistical computing package; http://www.r-project.org). As a control, HPKs and their cognate RRs (predicted based on genomic proximity) are strongly correlated within this same dataset (see Figure S1).

The difference in gene expression patterns and the domain shuffling both support the idea that these new paralogs have adopted new functional roles within the cell. It is not within the scope of this work to determine the environmental stimuli to which each HPK responds, yet some idea of the variety of possible direct or indirect signals can be inferred from Figure 9. For example, in cluster 1, a paralog with domain structure TM-PAS-PAS-PAS-HPK responds strongly to heat shock, and (to a lesser extent) nitrite stress, while a close paralog with domain structure TM-TM-HAMP-PAS-HPK responds most strongly to salt stress.

It is important to note that gene expression is an imperfect measure of function. Moreover, many HPKs may be expressed constitutively and regulated mainly at the level of phosphorylation. Nonetheless, we observe some clear cases in which expression is either upregulated or downregulated, and those trends are not generally conserved within these phylogenetic clusters. In some sense, signaling genes that are expressed under different sets of conditions could be considered to have different functions even if they regulated overlapping sets of genes. We feel that the general lack of coexpression, when combined with the diversity of newly evolved signaling domain architectures, together make a strong case for new functional roles.

### Genomic Distribution of HPK Families

**Figure 10:**
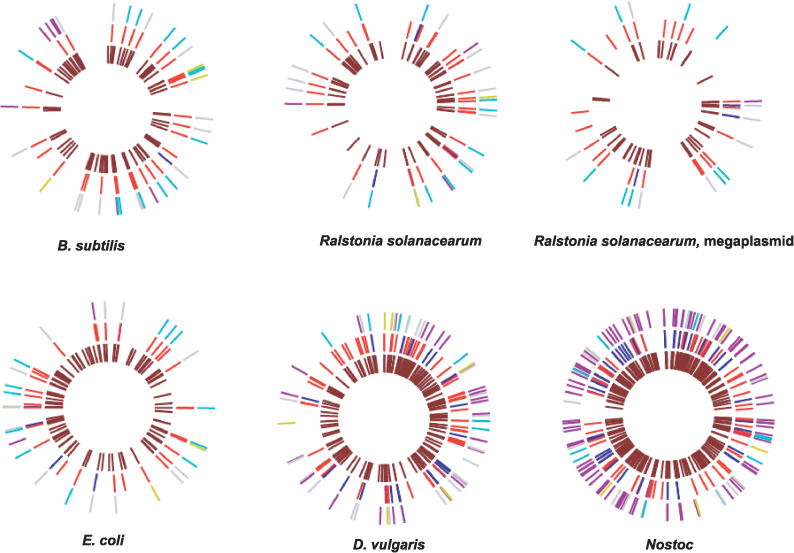
Genomic Distribution of HPKs and RRs The position of signaling proteins in several genomes is shown. In the outer ring, HPKs of different classes are shown: Old (gray), LSE (purple), HGT without duplication (blue), and genes that recently underwent a “Birth” event (green). The middle ring shows the position of response regulators, with blue colors indicating hybrid response regulators (containing HPK domains). The inner ring shows the location of all genes in each genome annotated as signaling proteins according to the MicrobesOnline database [37].

We looked at the distribution of paralogs within each genome to see if we could infer any information regarding the mechanism of gene family expansion. In *B. subtilis,* for example, all five of the *kin* genes are contained within a small region of the chromosome, with four of them very tightly spaced (the LSE or purple-colored genes in Figure 10). In general, however, we observed very little clustering of genes within genomes. To be more rigorous, we constructed a simple statistical test to measure clustering of new HPKs in a genome. We computed the distribution of nearest-neighbor distances between HPKs arising from LSE, and compared this with the distribution expected by chance (approximated by an exponential distribution with mean = [number of genes in genome] / [number of recent LSEs]). We then used the Kolmogorov-Smirnov test to determine if the two distributions were significantly different. Of the genomes we classified as having large numbers of LSEs, only *Nostoc* showed significant clustering. When we examined this result further, we identified the source of the clustering: a set of two adjacent HPKs, which likely work together to relay signals. The first gene in each of these pairs contains a wide variety of largely shuffled signaling domains, while the downstream gene contains a conserved HPK domain followed by a CheY-type regulator domain. After correcting for this by counting these adjacent pairs as a single duplication, we observed no clustering among LSE genes in *Nostoc.* Figure 10 shows an overview of genomic positions of HPKs and RRs across several species, none of which (apart from the *kin* locus of B. subtilis) appears to have significant clustering. Thus, the duplication of HPKs appears qualitatively different from the duplication of signaling domains within the N-terminal region of individual HPKs, as the latter often occur in long tandem stretches.

### Timing of Evolutionary Events

**Figure 11:**
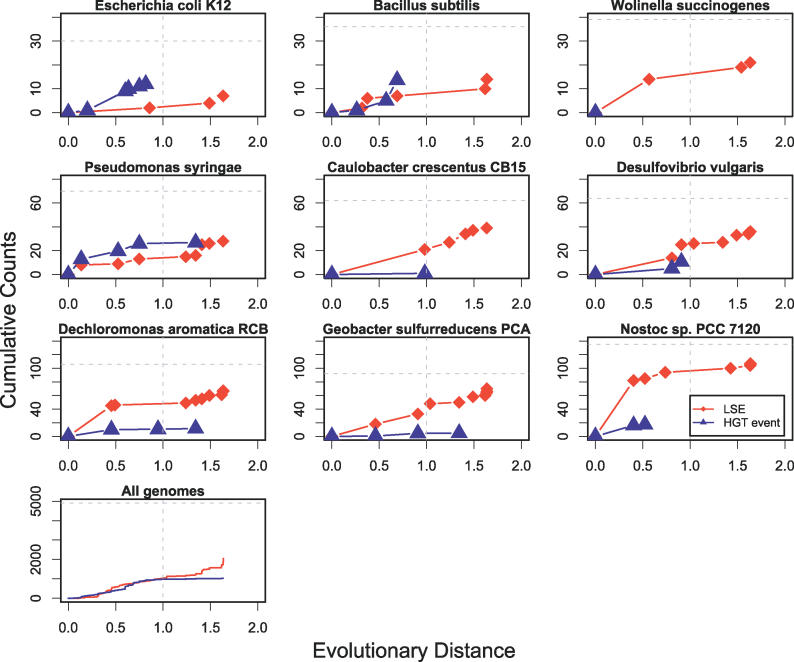
HPK Evolution over Time The number of HPKs entering a lineage is shown as a function of the time each HPK entered that lineage (i.e., the distance of that species to the last ancestor predicted to contain that HPK). Red lines/diamonds indicate LSE events, and blue lines/triangles indicate HGT events. Plots are cumulative showing all events dating more recently than the distance shown on the *x*-axis. HGT lines do not extend as far to the right as LSE lines, since they require genes to be lost from two consecutive outgroups. The vertical dashed line shows the phylogenetic cutoff distance, and the horizontal dashed line shows the total number of HPKs in each genome. Symbols indicate the evolutionary distance (arbitrary units; see Methods) of outgroups used in the analysis. Some clades with greater taxon sampling have better resolved timings. The last panel shows total numbers for all genomes.

Because our phylogenetic inference procedure identifies LSE and HGT events associated with a particular outgroup, we can trace the influx of HPKs into each lineage as a function of time. Figure 11 shows the number of HPKs predicted to have entered several lineages as a function of time (distance to divergence of outgroup). While different species here show different overall trends (some such as P. syringae gradually accumulated HPKs, while some such as *Nostoc* acquired most of their HPKs very recently), the species-averaged plot shows a steady influx of HPKs at a nearly constant rate back until about our phylogenetic cutoff distance of 1.0 (where HGT tends to saturate since it requires absence from at least two outgroups predating the transfer). Moreover, both HGT and LSE seem to be contributing at similar levels to the total number of HPKs, and both accumulate at about the same rate. It is important to note that the resolution of these figures depends directly on the number of sequenced bacterial groups at different levels of divergence from each genome, and caution should be used when comparing our evolutionary distances across distant taxa as differences in evolutionary rate were not rigorously modeled in this analysis. As more genome sequences become available, it will be possible to resolve the timing of these events with higher resolution, and even to measure turnover rates for HPKs.

## Discussion

### Different Strategies for Obtaining New Genetic Diversity

HPKs play a key role in allowing bacterial cells to sense and respond to their environment. We investigated specifically those HPKs that evolved recently because they are the most likely to shed light on how bacteria adapt to their particular niches. We investigated HGT and gene family LSE of HPKs. Among those genomes most likely to be highly regulated via environmental signals (i.e., those with the largest fraction of genes coding for HPKs), recently acquired genes came mostly from gene family expansion. Moreover, we found that gene family expansion is often accompanied by domain shuffling to produce signaling proteins with unique combinations of sensory domains, and that gene expression patterns among these paralogous genes were highly diverged. Together, these results suggest that gene family expansion and domain shuffling serve to facilitate the repurposing of existing signaling proteins to new tasks, but only in a subset of species. These species have adapted to their environment by trying new combinations of existing signaling domains to generate more novel genetic diversity. By contrast, the well-studied model organisms E. coli and B. subtilis likely acquire most of their new HPKs from existing bacteria, intact with identical sensory domains in many cases and together in operons with cognate response regulators.

### HGT and LSE across the Bacteria

Several recent studies [6,19–21] have begun to add detail to a framework for understanding the evolution of gene content in bacterial genomes. A key finding of the survey of gene acquisition/loss by Lerat and coworkers [19] is that gene family expansion is not a major mechanism of gene acquisition, at least in γ-proteobacteria. The current work focuses on a notable exception to this general rule, the HPKs. It is worth noting that in the set of genomes examined by Lerat and coworkers, HPKs did follow the general trend observed for other genes: new acquisitions generally represented an influx of new gene families rather than duplication of existing families. Therefore, it may be premature to conclude that it is this family of genes rather than the genomes themselves that are exceptional in this regard. Very large expansions of cytochrome genes have been reported for some of the δ-proteobacteria that we follow in this study [22], lending some support to the idea of genome-specific in addition to gene-specific differences in the propensity for expansion.

Whether or not some genomes have greater propensities for gene duplication overall, there are genome-specific differences in the likelihood of duplications in the HPK gene family. Expansion of HPKs is enriched in a subset of bacteria, primarily in early-branching proteobacteria. In contrast, the model organisms E. coli and B. subtilis rely largely on a set of ancient HPKs, and some HPKs recently acquired via HGT. As a result, the former group of bacteria contains a larger number of total HPKs, and their HPKs tend to have more unique combinations of upstream signaling domains.

### Alternative Explanations for HGT

LSE events are more straightforward to interpret than HGT events in our phylogenetic framework. Few would argue with the assertion that a gene present in multiple highly similar copies in a single clade, but only one (or zero) more distantly related copies in earlier-branching outgroups, reflects an LSE. Identification of HGT, on the other hand, relies on a less clear-cut argument: that a single HGT event is a more parsimonious (and therefore more likely) explanation for the absence of a gene in two or more consecutive outgroups (excluding reduced genomes) than is two or more consecutive deletion events. Therefore, some additional consideration of the evidence for HGT and alternative explanations for the observed patterns of gene distributions is called for.

One of the key lines of evidence supporting our approach for identifying HGT events is that we are able to identify donor–acceptor pairs with identical upstream signaling domains using only sequence data from the HPK domain. While this lends support to our domain-based BLASTp approach, it does not directly address the challenge of identifying a HGT scenario from multiple deletions, since vertically transmitted orthologs would also be likely to retain a similar set of upstream signaling domains. To test this hypothesis, we used a more conservative cutoff, absence from three consecutive outgroups, for identifying HGT events. This stricter cutoff produced nearly identical results (47.3% versus 47.4% of upstream domains were conserved using the two definitions), as reported in the earlier section on domain shuffling, lending support to a true difference between HGT and LSE genes. In addition, we found that horizontally transferred HPKs were *more* likely than vertically transmitted genes to occur in the same operon as a response regulator, supporting a distinction between these HGT genes and vertically transmitted genes.

We also examined the distribution of donor–acceptor genome pairs for the genomes with large amounts of inferred HGT events. While there was a general trend for HGT events within major bacterial groups (e.g., between B. subtilis and other firmicutes, or between E. coli and other proteobacteria), there was not a clear excess of transfer to and from a single pair of species, which might be expected if there were significant false positives resulting from an inaccurate species tree.

Finally, we observe that our results are in general agreement with a recently published study that estimated HGT rates across all gene families among sequenced bacteria [20]. B. japonicum has one of the highest rates of HGT among all bacteria in both studies, and most of the genomes indicated as having high rates of HGT in the previous study indeed are indicated to have significant levels of new HPKs arising through HGT in the present work. It should be noted that we did not describe results for Pirellula sp. in the current study because we could not resolve its phylogeny well enough to make confident assertions about its evolutionary history. Another difference between our study and the work by Ouzounis and coworkers [20] is our finding that the sequenced *Pseudomonas* species are outliers containing a large number of HPKs possibly acquired via HGT. *Pseudomonas* was also identified by Lerat et al. [19] as having recently acquired a large number of “ORFan” genes without obvious sequence homologs, but we did not see evidence for large numbers of novel HPK “birth” events. Whether *Pseudomonas* genomes confine their horizontal transfer to genes involved in signal transduction is an interesting question for further study.

### Mechanisms of HPK Evolution

The mechanisms that can lead to HGT of genes are well-studied. Phage, plasmids, and competence can all help facilitate the transfer of foreign DNA. In a dramatic example, most of the HPK genes (10 of 17) on the megaplasmid of R. solanacearum are predicted to be horizontally transferred into that lineage, and nine of ten of these are inferred to have transferred very recently (i.e., they are absent in the closest two outgroups). This suggests the megaplasmid may have served as a vessel for the import of these genes (see plasmid genes in Figure 10). In contrast, only 11 of 27 genes on the main chromosome are inferred to be the result of horizontal transfer, and of these, only five are absent in the closest outgroups.

Despite the fact that LSE is a major driving force in the genesis of new HPKs (by our estimates, a larger factor than HGT), the mechanism(s) behind these expansions are unclear. In some cases, transposons can be implicated in recent gene expansions, in particular when the transposase genes occur next to the gene copies undergoing expansion. Yet, for most of the large expansions we observed, there was no evidence of nearby transposons, and duplications of HPKs were accompanied by domain shuffling of their upstream regions. Transposons are unsatisfying as an explanation in this case because new combinations of upstream signaling domains are not created directly from single transposition events (an insertion event would interrupt the reading frame between the HPK domain and the new signaling domain). Thus, genomes involving large LSEs may contain additional mechanisms that allow for the rearrangement of individual domains, and the presence of these mechanisms may account for the predisposition of those genomes to LSE rather than to HGT.

A recent genomic survey of recombination machinery across bacteria did not identify specific components unique to this set of genomes [23]. Instead, many of the species we identified as rich in gene expansions were highlighted as genomes known to undergo recombination, but lacking a full complement of presynaptic recombination proteins. Perhaps some of these genomes contain alternative, yet-to-be-identified presynaptic genes with properties that facilitate LSE and/or domain shuffling. Gene duplication is known to happen at high frequency near the ends of the linear *Streptomyces* chromosome [11,24], but probably through a different mechanism than other LSE-rich genomes because the domain structure and cognate response regulators are more highly conserved in this species compared with the other species prone to duplication events.

An alternative to the theory of genome-specific recombinational machinery is a role for phage or other extrachromosomal elements in the duplication and rearrangement of domains in these signaling proteins. A recent study by Aravind and coworkers found massive domain shuffling and LSE of a family of fungal transcription factors occurring inside viral genomes [25]. A phage origin for LSEs would also explain the discrepancy between the relatively common tandem duplication and shuffling of signaling domains within HPKs (which could occur in the phage genome), and the apparent random positioning of paralogous HPKs across the genome (which would be the result of random phage insertions). To test the phage origin hypothesis, we examined large LSEs across a number of genomes, but did not find a statistically significant excess of phage genes near paralogs. Moreover, a BLASTp search of phage databases yielded only a few very weak hits to HPKs.

Whether genome-specific recombinational machinery, extrachromosomal elements, or other factors (such as environments that select for large numbers of HPKs) are responsible for generating genetic diversity among HPKs, it is clear that there are genome-specific differences. Genomes such as *Streptomyces* represent still a different paradigm (i.e., capability for large-scale expansion but little new diversity, at least at the level of domain structure). Why this creative ability appears to be limited to a subset of genomes, and whether these differences among taxa extend to other gene families, emerge as key directions for future research.

## Materials and Methods

### Inferring evolutionary histories of HPKs.

To describe the origin of HPK domains, we examined the phylogenetic distribution of those domains across other taxa. Similar to previous work [5,6,20,26], we inferred “birth” events when a domain was found only in a single clade and horizontal transfer events in cases where multiple gene losses were needed to explain the phylogenetic distribution, assuming vertical descent. Unlike the previous studies mentioned, we were interested in cataloging and determining the relative age of gene duplication events. Because different HPK families can be considered ancient paralogs, we were not able to use a simple presence/absence criterion to describe the distribution of genes in taxa. Instead, we used a BLASTp score cutoff on sequence similarities to infer the phylogenetic distributions of particular subfamilies as described in the following section. Importantly, our analysis was based only on the histidine kinase domains (on average, 224 residues), excluding sensory and other domains, so that we could discriminate the independent processes of domain evolution and shuffling of upstream signaling domains.

The inference process is briefly outlined below (details follow in later sections). (1) Construct phylogeny for all species using concatenated ubiquitous genes. (2) Identify HPKs from coding regions of all genomes. (3) Parse HPKs into domains (PAS, HAMP, TM, HPK, etc.). (4) Find best hits of each “query” HPK (domain) to outgroups: move up one node in the species tree (starting from the genome containing the “query” HPK). Next, add the leaves of the new branch to the current “outgroup” (if the current node has a bootstrap value of less than 80%, then move up one node in the species tree again), and record the best hit of the “query” HPK to the current “outgroup” species. Next, record the number of “paralog” HPKs (in the same genome as the query) that have higher BLASTp scores than the best hit to the current outgroup. The gene count at this node [see step 6] is equal to [1/number of paralogs]. The duplicate count at this node [see step 6] is equal to [1 – (the gene count)]. Repeat with a new “outgroup” until the root node is reached. (5) Build presence/absence profile from best hits: if the best hit to any outgroup is less than 25% of the BLASTp score of the query HPK to itself, then set that outgroup to zero (for absent); otherwise, starting from the most ancient outgroup *(Aquifex aeolicus),* set the score of each outgroup equal to one (for present) unless the best hit to that outgroup is less than the best hit to an older outgroup by a bitscore value of 20 (in which case set the score to zero for absent). If any outgroup with score zero contains only reduced genomes with fewer than ten HPKs, change its score to two [for unknown]. (6) Infer evolutionary history from presence/absence profile—if there are two or more consecutive zeroes (ignoring twos) in the presence/absence profile for a given HPK, then it is considered HGT. The oldest outgroup containing a one before the run of zeroes is considered the age of the HGT event; if this age is past the phylogenetic cutoff, then the HGT is not counted. The gene count at these nodes is added to the number of HGT events for the query genome—otherwise, if there are no ones in outgroups older than the phylogenetic cutoff distance, then it is considered a HPK subfamily “birth,” and the gene event score at the oldest “one” is added to the estimated number of “birth” events. The number of gene duplications is estimated by adding the duplicate count at the oldest outgroup containing a “one” that is within the phylogenetic cutoff age to the total LSE counts.

### Step 1: Construction of bacterial species tree.

Each gene that was present in every bacterial genome studied, without obvious paralogs (no other genes in the same cluster of orthologous groups), was used to construct a species tree. The tree was built based on the multiple sequence alignment using the concatenated sequences of these 15 genes, which are listed in Table S1. Muscle [27] was used for the multiple sequence alignment; gaps were trimmed using MEGA3 [28]. A neighbor-joining tree with 100 bootstrap replicates was built using the Phylip [29] software package with the PMB (probability matrix from blocks) [30] amino acid substitution model. Gamma-distributed rates were used with a shape parameter of 0.72, which was estimated using TREE-PUZZLE [31]. Branch lengths were then constructed using the bootstrap consensus tree topology and the PROTDIST program included in the PHYLIP package [29], enforcing equal distances of each species to the tree root. Unequal rates for different taxa were not taken into consideration, and therefore distances should not be interpreted as accurate estimates of evolutionary time. The tree is included in Dataset S3 for review.

Our species tree is based on concatenated protein (mostly ribosomal) genes. Similar approaches have been shown to be effective at producing well-resolved trees [9,10]. We also experimented with 16S rDNA sequence trees and genome content trees, but found that the concatenated gene trees produced better-resolved trees that seemed to avoid some unexpected groupings that probably arise from differences in GC content and reduced genomes. We include our species tree in Newick format as Dataset S3 for review. We chose the root of our tree at the last common ancestor of A. aeolicus and other bacteria. We do not claim strong evidence for the early branching of this lineage; instead, we limit our analysis to more recent evolutionary events that do not depend strongly on the topology of the deepest-branching nodes.

### Step 2: Identification of HPKs.

We identified 4,959 HPKs in 20 archaeal and 187 bacterial genomes based on sequence and profile similarities. A protein was considered a putative HPK if the protein contained a histidine kinase domain, (IPR005467 as measured using the InterPro software suite [32]), or was assigned to the signal transduction histidine kinase COG4582 using a profile-based RPS-BLAST search [33,34]. In addition, we considered all proteins that were not picked up by either method, but that contained an ATPase domain according to SuperFamily motif SSF55874 [35]. If a protein contained an ATPase domain and was a transmembrane protein or had one or more known signaling domains (e.g., PAS, HAMP), then it was also considered an HPK.

For 123 of 130 genomes, our approach compared favorably with that of Galperin [2], who used manually curated PSI-BLAST searches to identify likely HPKs, in that we identified nearly the same number of HPKs. For seven genomes, the total counts differed by more than four, with S. coelicolor being the largest deviation (the study by Galperin identified 95, while our method found only 81). Overall, we argue that our main findings are not substantially affected by the differences in the numbers of HPKs, as our overall numbers are consistent with the manually curated study by Galperin. A detailed comparison on a genome-by-genome basis is provided as Dataset S1. A FASTA format file including all HPK domains used in this analysis is included as Dataset S4.

No HPK domains were found in sequenced genomes within the taxonomic group Mollicutes. Bacterial genomes with small genome size (i.e., <1,000 protein-coding genes) tended to have few or no HPKs, and the proportion of HPKs was correlated with the genome size. Approximately half (11 of 20) of the archaeal genomes with a large range of genome sizes (553–3,106 protein-coding genes) had no HPKs. HPKs from archaeal genomes were not considered in this analysis.

### Step 3: Identification of structural and signaling domains in histidine kinases.

We used the profile-based methods included in the InterPro software suite [32] to identify common signaling domains, and TmHMM [36] to predict transmembrane regions of HPKs. The key protein families used were: CheY-like response regulator receiver: PF00072, SSF52172; CheB methylesterase: PS50122; CheR-type MCP methyltransferase: PS50123; PAS: SM00091, SSF55785, or TIGR00229; HAMP: PF00672, PS50885; GAF: SSF55781, SM00065; Hpt: SSF47226, PF01627; CACHE: PF02743; Phytochrome, light-sensing: PF00360, PS50046 (PF, Pfam; SM, SMART; SSF, SuperFamily; TIGR, TIGRFAM; PS, PROSITE PROFILE).

### Step 4: Build best BLASTp hit profile.

The first step in this procedure was to identify a set of unambiguous “outgroups” for each species. We considered each ancestor node on the tree built in step 1 that contained the target species, starting with the most recent. If the bootstrap support at that node was at least 80%, then all the species present in the leaves of the new branch were considered to constitute an “outgroup.” If the bootstrap support was less than 80%, then those species were put aside until an internal node with at least 80% support was reached, and all of the leaves of those new branches were combined into a single “outgroup.” The best BLASTp hit to any of the HPK domains contained within each outgroup was recorded.

### Step 5: Build presence/absence profile from best hits.

Each best-hit profile was converted to a string of integers (0, 1, or 2), indicating whether the particular gene subfamily was likely to be present or absent from each outgroup. First, a lower boundary was placed on BLASTp hits: scores less than 25% of the maximal BLASTp score (of the query gene to itself) were not considered, as these are essentially different subfamilies of HPKs. These low scores were set to zero.

Outgroups that had BLASTp hits greater than the best hit to any more distant outgroups (minus a threshold bitscore of 20 from the distant outgroup hit) were assigned a one. A string of ones, therefore, indicates a set of BLASTp distances consistent with vertical inheritance of the query HPK. The threshold of 20 makes our method more conservative in calling gene absences, and was intended to lessen the effects of evolutionary rate differences among lineages and possible errors in our species tree topology.

Outgroups without a hit greater than that seen in older groups were assigned zero, unless every genome in the outgroup had less than ten HPKs. In that case, it is likely that those species had undergone a genome-wide reduction in the number of HPKs, and multiple absences from such outgroups does not provide strong support for the alternative hypothesis of HGT. These outgroups were assigned a value of two. The cutoff of ten HPKs is essentially an ad hoc rule that works well at identifying reduced genomes in the set of genomes studied. A list of excluded genomes is given in Dataset S2. A website including all the raw BLASTp scores, their presence/absence profiles, and other key information for each HPK in this study is provided at http://microbesonline.org/hpk.

### Step 6: Inferring evolutionary events from presence/absence profiles.

We sought to identify the events that led to the complement of HPKs observed in each genome contained in the MicrobesOnline database [37] as of February 2005. We considered four possible origins for extant HPKs: (1) duplication of pre-existing HPKs; (2) horizontal transfer from distantly related genomes; (3) “birth” of novel HPK subfamilies; and (4) “old” HPKs that were present in the genome early in its evolutionary history.

*Phylogenetic cutoff.* To classify evolutionary events as “recent,” we defined a phylogenetic cutoff distance before which we did not report events. This had the additional benefit of making our analysis robust to the topology of the deepest and most-difficult-to-resolve branches. The cutoff distance used is roughly equivalent to the divergence time of E. coli from the most distantly related γ-proteobacteria. For details on the cutoff for different lineages, see the tree and the Web site provided at http://microbesonline.org/hpk. Because different taxa have different evolutionary rates for the same set of genes, we used a cutoff based on a species tree in which the distance of each leaf to the root is assumed to be equal. We used this “linearized” tree distance not to compute accurate divergence times, but to enforce our phylogenetic cutoff distance across different lineages more evenly.

*“Event” counting.* The basic algorithm for counting HGT, LSE, and birth events is discussed above, but some explanation is necessary for the “gene count” and “duplicate count” calculations. Because we consider each HPK independently, we need to adjust the counts such that a gene that is horizontally transferred once, and subsequently duplicated, is not counted as two HGT events. For this reason, we count only (1/number of paralogs) HGT events for each duplicate copy. By similar reasoning, a gene duplicated four times should not be counted as five duplication events, because one copy represents the original gene. To avoid overcounting of duplication events, we count only [1 – (1/number of paralogs)] LSE events for each paralog.

*Gene expression data.* Gene expression microarray data was downloaded from the MicrobesOnline database [37]. A full description of the salt, heat, and nitrite stress experiments is given in [38–40]. Briefly, all experiments were performed in LS4D (lactate–sulfate) medium under anaerobic conditions. Cells were grown to log phase and then subject to stressors: 8 °C cold shock (30 °C control), 50 °C heat shock (37 °C control), 1,000 ppm oxygen, 500 mM NaCl, and 2.5 mM nitrite. Each sample was measured relative to a genomic DNA control, and reported values are the log-ratio of expression levels at the indicated timepoint versus the pre-stress (0 min) levels for the same biological sample.

## Supporting Information

Dataset S1 Detailed Raw and Processed Data for Each Genome (138 KB XLS)

Dataset S2 Genomes Excluded from HGT Calculation (12 KB XLS)

Dataset S3 Species Tree with Distances and Bootstrap Scores (4 KB TXT)

Dataset S4 FASTA Format File Including All HPK Domain Sequences Used (3.0 MB TXT)

Figure S1 Correlation in Expression between Operons and RRs (30 KB DOC)

Table S1 Clusters of Orthologous Groups Used to Build Species Tree (21 KB DOC)

### Accession Numbers

The Microbes Online (http://microbesonline.org) accession numbers for the paralogs discussed in this paper are DVU0025 (208953) and DVU0737 (206163).

## Abbreviations

HGT: horizontal gene transfer
HPK: histidine protein kinase
LSE: lineage-specific expansion
RR: response regulator
