# Supplementary material for: The Evolution of Two-Component Systems in Bacteria Reveals Different Strategies for Niche Adaptation
Source: PLoS Comput Biol. 2006 Nov 3;2(11):e143. doi: 10.1371/journal.pcbi.0020143 (PMC1630713; doi:10.1371/journal.pcbi.0020143)
Supplement: Supplementary file 1 [file pcbi.0020143.sg001.doc]

**Supplementary Figure.**

*Correlation of HPKs and co-operonic RRs in* D. vulgaris. Histogram of correlation between co-operonic HPK-RR pairs compared to the histogram for randomly chosen pairs. Pairs on the same operon show significantly higher correlation than random pairs, and higher correlation than observed for paralogous HPK clusters in *D. vulgaris*.
