# Supplementary material for: The Evolution of Two-Component Systems in Bacteria Reveals Different Strategies for Niche Adaptation
Source: PLoS Comput Biol. 2006 Nov 3;2(11):e143. doi: 10.1371/journal.pcbi.0020143 (PMC1630713; doi:10.1371/journal.pcbi.0020143)
Supplement: Supplementary file 2 [file pcbi.0020143.st001.doc]

**Supplementary Table.**

List of genes used to build species tree:

| COG202 | DNA-directed RNA polymerase, alpha subunit/40 kD subunit [Transcription] |
| --- | --- |
| COG533 | Metal-dependent proteases with possible chaperone activity [Posttranslational modification, protein turnover, chaperones] |
| COG536 | Predicted GTPase [General function prediction only] |
| COG12 | Predicted GTPase, probable translation factor [Translation, ribosomal structure and biogenesis] |
| COG216 | Protein chain release factor A [Translation, ribosomal structure and biogenesis] |
| COG244 | Ribosomal protein L10 [Translation, ribosomal structure and biogenesis] |
| COG197 | Ribosomal protein L16/L10E [Translation, ribosomal structure and biogenesis] |
| COG256 | Ribosomal protein L18 [Translation, ribosomal structure and biogenesis] |
| COG292 | Ribosomal protein L20 [Translation, ribosomal structure and biogenesis] |
| COG211 | Ribosomal protein L27 [Translation, ribosomal structure and biogenesis] |
| COG92 | Ribosomal protein S3 [Translation, ribosomal structure and biogenesis] |
| COG49 | Ribosomal protein S7 [Translation, ribosomal structure and biogenesis] |
| COG103 | Ribosomal protein S9 [Translation, ribosomal structure and biogenesis] |
| COG691 | tmRNA-binding protein [Posttranslational modification, protein turnover, chaperones] |
| COG195 | Transcription elongation factor [Transcription] |
